# The Alterations of Mitochondrial Function during NAFLD Progression—An Independent Effect of Mitochondrial ROS Production

**DOI:** 10.3390/ijms22136848

**Published:** 2021-06-25

**Authors:** Inês C. M. Simões, Ricardo Amorim, José Teixeira, Agnieszka Karkucinska-Wieckowska, Adriana Carvalho, Susana P. Pereira, Rui F. Simões, Sylwia Szymanska, Michał Dąbrowski, Justyna Janikiewicz, Agnieszka Dobrzyń, Paulo J. Oliveira, Yaiza Potes, Mariusz R. Wieckowski

**Affiliations:** 1 Nencki Institute of Experimental Biology of Polish Academy of Sciences, 02-093 Warsaw, Poland; i.simoes@nencki.edu.pl (I.C.M.S.); m.dabrowski@nencki.edu.pl (M.D.); j.janikiewicz@nencki.edu.pl (J.J.); a.dobrzyn@nencki.edu.pl (A.D.); 2 CNC—Center for Neuroscience and Cell Biology, CIBB—Center for Innovative Biomedicine and Biotechnology, University of Coimbra, 3004-504 Coimbra, Portugal; uc2017238707@student.uc.pt (R.A.); jose.teixeira@uc.pt (J.T.); uc2012144200@student.uc.pt (A.C.); pereirasusan@gmail.com (S.P.P.); ruifmsimoes@gmail.com (R.F.S.); pauloliv@ci.uc.pt (P.J.O.); 3 CIQUP/Department of Chemistry and Biochemistry, Faculty of Sciences, University of Porto, 4169-007 Porto, Portugal; 4 Department of Pathology, The Children’s Memorial Health Institute, 04-730 Warsaw, Poland; A.Karkucinska-Wieckowska@ipczd.pl (A.K.-W.); s.szymanska@ipczd.pl (S.S.); 5 Laboratory of Metabolism and Exercise (LametEx), Research Centre in Physical Activity, Health and Leisure (CIAFEL), Laboratory for Integrative and Translational Research in Population Health (ITR), Faculty of Sport, University of Porto, 4200-450 Porto, Portugal

**Keywords:** non-alcoholic fatty liver, hepatic disease progression, metabolism, oxidative stress, liver autophagy

## Abstract

The progression of non-alcoholic fatty liver (NAFL) into non-alcoholic steatohepatitis implicates multiple mechanisms, chief of which is mitochondrial dysfunction. However, the sequence of events underlying mitochondrial failure are still poorly clarified. In this work, male C57BL/6J mice were fed with a high-fat plus high-sucrose diet for 16, 20, 22, and 24 weeks to induce NAFL. Up to the 20th week, an early mitochondrial remodeling with increased OXPHOS subunits levels and higher mitochondrial respiration occurred. Interestingly, a progressive loss of mitochondrial respiration along “Western diet” feeding was identified, accompanied by higher susceptibility to mitochondrial permeability transition pore opening. Importantly, our findings prove that mitochondrial alterations and subsequent impairment are independent of an excessive mitochondrial reactive oxygen species (ROS) generation, which was found to be progressively diminished along with disease progression. Instead, increased peroxisomal abundance and peroxisomal fatty acid oxidation-related pathway suggest that peroxisomes may contribute to hepatic ROS generation and oxidative damage, which may accelerate hepatic injury and disease progression. We show here for the first time the sequential events of mitochondrial alterations involved in non-alcoholic fatty liver disease (NAFLD) progression and demonstrate that mitochondrial ROS are not one of the first hits that cause NAFLD progression.

## 1. Introduction

Non-alcoholic fatty liver disease (NAFLD) affects around 24% of the worldwide population. It is expected that numbers will rise in parallel with the prevalence of obesity and type 2 diabetes mellitus, known as risk factors for the onset and the progression of NAFLD [1,2]. Therefore, the accumulation of fat in the liver (simple steatosis or non-alcoholic fatty liver, NAFL) can progress to inflammatory steatohepatitis (NASH), a condition in which a pro-inflammatory state may lead to the activation of Kupffer and stellate cells that stimulate collagen deposition and liver fibrosis [3]. Ultimately, liver cirrhosis may culminate in hepatocellular carcinoma [4].

During an early NAFL stage, there are multiple hepatic adaptations to regulate fat accumulation caused by the continuous inflow of fatty acids (FAs) to the liver [5]. Upregulation of mitochondrial activity (including fatty acid oxidation (FAO), tricarboxylic acid (TCA) cycle, and oxidative phosphorylation (OXPHOS)) was reported in NAFLD non-obese/obese patients, as well as in animal models [5,6]. In this state, the mitochondrial electron transport chain (ETC) can be a major source of superoxide anion (O_2_^•−^) and hydrogen peroxide (H_2_O_2_) production [7], which, along with a deregulated mitochondrial antioxidant defense system, cause a pro-oxidant state [8,9,10]. Notably, several authors demonstrated that the metabolic flexibility of mitochondria is lost along with disease progression [11,12], oxidative stress being pointed out as the main contributor of mitochondrial dysfunction characterized by decreased carnitine palmitoyl transferase-1α (CPT-1α), OXPHOS complexes activities and respiration, high proton leak rate, and development of insulin resistance in NASH [10,13]. Interestingly, a correlation between mitochondrial dysfunction, insulin resistance, and high tumor necrosis factor-α (TNF-α) levels suggests that progressive mitochondrial impairment determines the development of hepatic inflammation [14].

Irrespective of the fact that the mechanisms underlying the link between oxidative stress and mitochondrial dysfunction are complex, recently, it has been questioned whether mitochondrial-derived reactive oxygen species (ROS) are the cause or an accompanying event of mitochondrial dysfunction in NAFLD. This question is in line with the findings of Einer and colleagues, who reported no signs of mitochondrial oxidative stress during mitochondrial adaptation in steatotic mice [15]. Moreover, these observations were confirmed by our recent study, in which we proposed the involvement of peroxisomes as an extra-mitochondrial source of ROS in hepatic oxidative injury in a mouse model of NAFLD [16].

In order to answer this question, the present study intended to clarify the role of mitochondrial functional alterations and ROS production along with disease progression from NAFL to a more advanced disease stage. Using our previously established NAFLD mouse model [16], the present work shows that mitochondrial remodeling and respiratory alterations are independent of mitochondrial ROS production. Moreover, we demonstrate that the convergence of the hepatic oxidative damage, probably induced in a peroxisomal FAO-dependent manner, and the failure of the autophagic degradation system are tightly correlated with NAFLD severity and progression.

## 2. Results

### 2.1. WD Induces Animal Body Weight Gain, Fatty Liver Accumulation with Signs of Liver Damage, Ballooning, and Fibrosis

**Figure 1:**
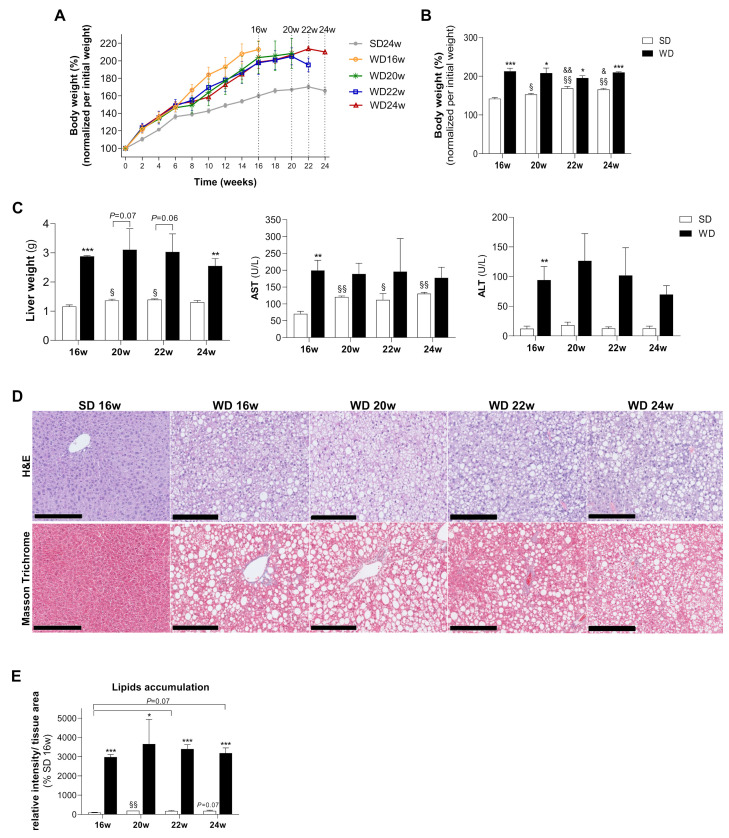
Western diet (WD) causes body weight gain concomitant with hepatic steatosis and damage along 24 weeks of feeding when compared with standard chow diet (SD). (**A**) Body weight gain profile along 16, 20, 22, and 24 weeks of WD feeding. (**B**) Body weight at the correspondent euthanasia time. (**C**) Wet liver weight at the euthanasia time and aspartate aminotransferase (AST) and alanine aminotransferase (ALT) plasmatic activity levels. (**D**) Representative images of paraffin-embedded liver sections with hematoxylin and eosin (H&E) and Masson Trichrome stains. Scale bar, 250 µm; 10× magnification. (**E**) Neutral lipids levels were obtained from the quantification of H&E staining of three independent images per animal and condition, being data expressed in % of SD16w group. Number of replicates = 3. All data are expressed as the mean ± SEM. (*) vs. SD, (§) vs. 16 w, (&) vs. 20 w (*p* < 0.05); (**) vs. SD, (§§) vs. 16 w, (&&) vs. 20 w. (*p* < 0.01); (***) vs. SD (*p* < 0.001); *p* values were determined using two-way ANOVA.

Feeding mice with a Western diet induced an increase of 25–50% in body weight and 100–200% in liver weight during the 16, 20, 22, and 24 weeks-feeding period (Figure 1A–C; Supplementary Figure S1A,B). Increased levels of the plasma enzymes ALT and AST reported hepatic damage (Figure 1C). Moreover, WD induced steatosis, although the accumulation of lipids occurred at a similar extent in all time points studied (Figure 1D,E). These results suggest that from 16 until 24 weeks, WD animals reached their maximal capacity to accumulate lipid droplets in hepatocytes. Moreover, our WD-induced mouse model of NAFLD is associated with higher hepatocyte ballooning in comparison with SD-fed groups. Moreover, NAS score evaluation revealed an absence of inflammatory markers and no significant alterations of fibrosis in WD-fed groups (Supplementary Figure S2A,B). These findings indicate an incomplete progression of NAFL into the more severe stage of NAFLD in animals fed for 24 weeks with WD.

### 2.2. Increased Accumulation of Triglycerides and Cholesteryl Esters in Hepatocytes Triggered by WD

**Figure 2:**
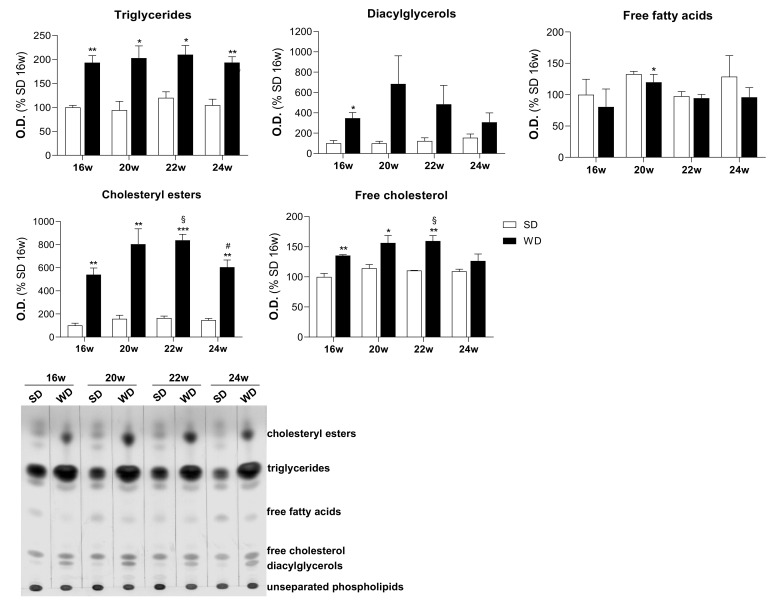
Fatty liver is enriched in triglycerides (TGs) and cholesteryl esters (CEs). Neutral lipids accumulation in TGs, diacylglycerols (DAGs), free fatty acids (FFAs), CEs, and free cholesterol (Chol) and representative thin layer chromatography image. Number of replicates = 3. All data are expressed as the mean ± SEM. (*) vs. SD, (§) vs. 16 w, (#) vs. 22w (*p* < 0.05); (**) vs. SD (*p* < 0.01); (***) vs. SD (*p* < 0.001); *p* values were determined using two-way ANOVA. SD, standard chow diet; WD, Western diet.

Typically, WD-fed mice exhibit hepatic steatosis characterized by the accumulation of neutral lipids. By using TLC, we demonstrated that this accumulation is enriched in triglycerides (TGs) (2-fold) and cholesteryl esters (CEs) (≥6-fold) after 16, 20, 22, and 24 weeks of WD-feeding versus SD-feeding regimen (Figure 2). We observed here increased levels of diacylglycerols (DAG) and free cholesterol (Chol) up to 24 weeks of feeding, as a result of stimulation of anabolic and catabolic lipid metabolism, in agreement with our previous work [16]. No major alterations were observed for free fatty acids levels at the studied time points (Figure 2). The distinct signature of neutral lipid accumulation between the SD- and the WD-fed groups was confirmed by the principal component analysis (PCA) represented in the Supplementary Figure S3.

### 2.3. Progressive Decline of Mitochondrial Function in the WD-Induced Mouse Model of NAFLD

**Figure 3:**
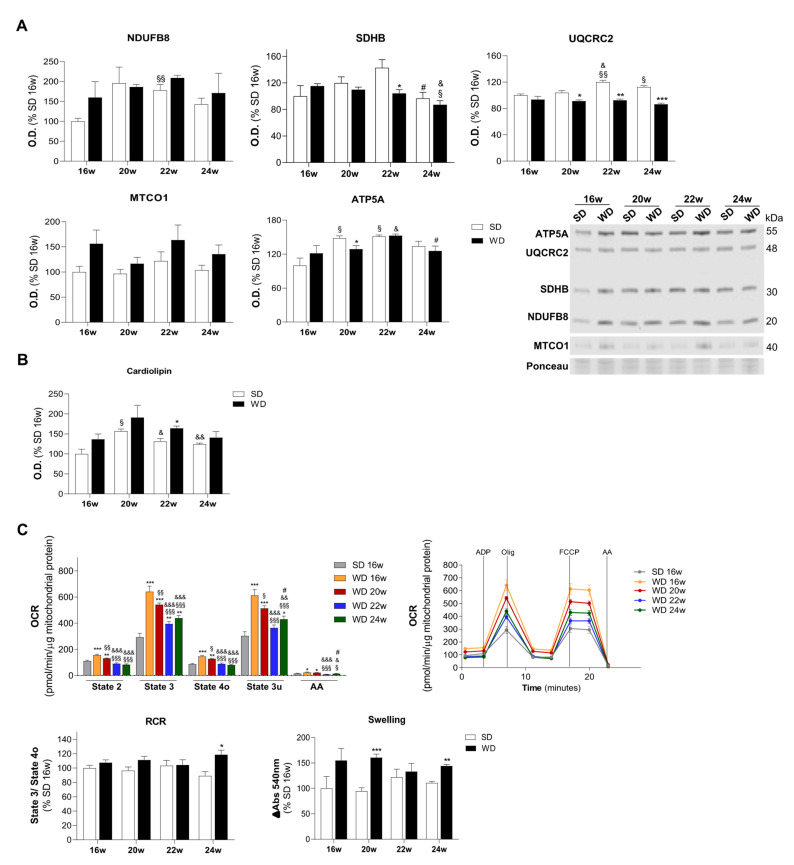
WD feeding induces a higher hepatic mitochondrial oxygen consumption rate (OCR) at 16 weeks, an effect that was decreased for longer timepoints and accompanied by mitochondrial permeability transition pore (MPTP) opening. (**A**) OXPHOS subunits levels (NDUFB8, complex I; SDHB, complex II; UQCRC2, complex III; MTCO1, complex IV; ATP5A, ATP synthase) expressed as optical density (arbitrary units), with Ponceau being used as a loading control. Representative image of OXPHOS subunits Western blot. (**B**) Cardiolipin levels present in liver mitochondria. (**C**) OCR of CII-linked hepatic mitochondrial respiration supported by succinate (left panel); and a representative profile of OCR (right panel); Respiratory control ratio (RCR) (state 3/state 4o) and opening MPTP—swelling (lower panel). Number of replicates = 3. All data are expressed as the mean ± SEM. (*) vs. SD, (§) vs. 16 w, (&) vs. 20 w, (#) vs. 22 w (*p* < 0.05); (**) vs. SD, (§§) vs. 16 w, (&&) vs. 20 w (*p* < 0.01); (***) vs. SD, (§§§) vs. 16 w, (&&&) vs. 20 w (*p* < 0.001); *p* values were determined using two-way ANOVA. AA, antimycin A; FCCP, carbonyl cyanide-p-trifluoromethoxyphenylhydrazone; Olig, oligomycin; SD, standard chow diet; WD, Western diet.

Mitochondria are considered the prominent cellular organelles responsible for the FAs oxidation, essential to overcome the excess of FAs inflow/accumulation inside hepatocytes [5]. In order to characterize mitochondrial function under such circumstances, we first evaluated protein expression of OXPHOS complexes and mitochondrial lipids after 16, 20, 22, and 24 weeks of feeding mice with WD, by using mitochondria fraction. Analysis of OXPHOS subunits levels showed an upward trend for complex I subunit (NDUFB8), complex II subunit (SDHB), complex IV subunit (MTCO1), and ATP synthase subunit (ATP5A) in WD-fed groups at 16th week. However, WD feeding caused a time-dependent decrease of complex III subunit (UQCRC2) and ATP5A at the 20th week, SDHB and UQCRC2 at 22nd week, and UQCRC2 at 24th week (Figure 3A). Additionally, an upward trend of cardiolipin and phosphatidylinositol (PI) levels for WD-fed groups compared with SD was found, this difference being statistically significant for the 22nd week. No alterations were found in other mitochondrial phospholipid species (Figure 3B; Supplementary Figure S4A). We next investigated mitochondrial bioenergetic capacity to reveal whether the observed OXPHOS remodeling and lipid profile alterations resulted in altered mitochondrial function. We evaluated mitochondrial oxygen consumption rate (OCR) in the hepatic mitochondrial fractions by using succinate as a substrate. Given that no major significant differences in OCR values of all respiratory states were found between SD-feeding time points (Supplementary Figure S4B), to simplify the interpretation of the data, we represent OCR values of all WD-fed mice groups in comparison with the SD16w group. Interestingly, at 16 and 20 weeks of WD feeding, significantly increased OCR values were observed in basal mitochondrial respiration (State 2), ADP-stimulated respiration (State 3), non-ADP-simulated respiration (State 4o), maximal respiration (State 3u), and after antimycin-A addition in comparison with the SD group. However, this increase was followed by a progressive and significant decrease of OCR for longer WD-feeding timepoints (WD22w and WD24w groups). The respiratory control ratio (RCR), which represents the mitochondrial coupling state, did not show significant differences between WD- and SD-fed groups at 16, 20, and 22 weeks, but a significant increase of RCR in WD24w was indicative of a lower proton leak and/or a higher mitochondrial phosphorylation capacity from complex II at this time point (Figure 3C). Additionally, electron flow assay showed a higher transport of electrons through complex I, II, and III in the WD20w and just through complex II in the WD22w and WD24w groups (Supplementary Figure S5A). Again, no major significant differences were observed between all SD-feeding time points tested (Supplementary Figure S5B). Furthermore, isolated mitochondria from WD-fed mice were more susceptible to initiate the calcium-sensitive permeability transition pore. Indeed, when mitochondria were exposed to swelling-promoting conditions, a higher susceptibility for the opening of MPTP in livers of mice fed for 20 and 24 weeks with WD was found (Figure 3C, lower right panel). These alterations allow the discrimination of WD20, 22, and 24 w groups from the SD condition, as visualized in the PCA analysis (Supplementary Figure S5C). Altogether, increased OXPHOS subunits expression and higher cardiolipin levels (Figure 3A,B) support the higher oxygen consumption rate at 16 weeks, alterations along time possibly being involved in the higher sensitivity of mitochondria to MPTP opening after 20 weeks of WD feeding.

### 2.4. Declined Mitochondrial ROS Generation Is Associated with NAFLD Progression

**Figure 4:**
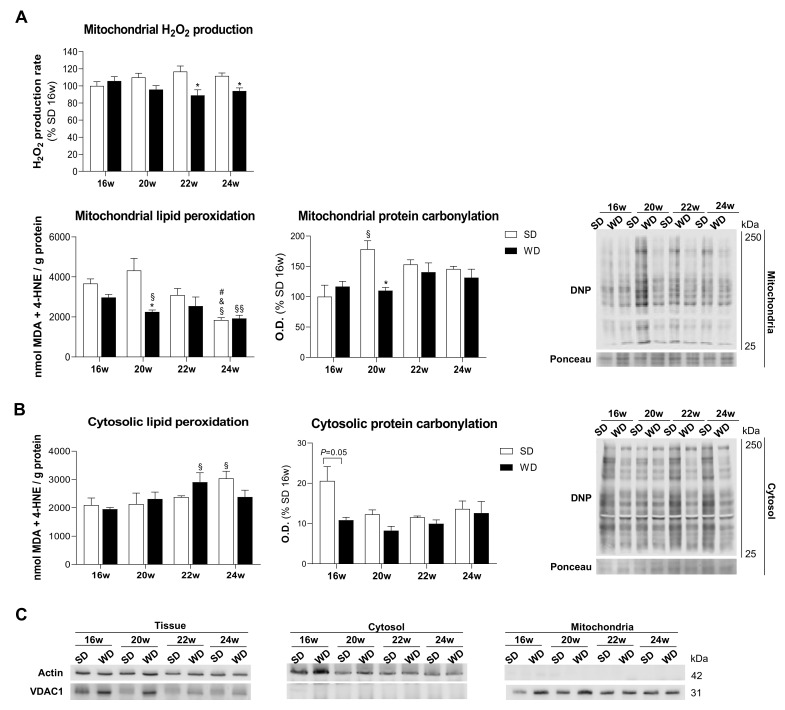
Western diet (WD) induces a decrease in hepatic mitochondrial reactive oxygen species (ROS) production along non-alcoholic fatty liver disease (NAFLD) progression. (**A**) Mitochondrial H_2_O_2_ production rate, lipid peroxidation (MDA and 4-HNE) levels, and protein carbonylation (DNP) levels. Representative image of mitochondrial protein carbonylation (DNP) levels. Ponceau was used for data normalization. (**B**) Cytosolic lipid peroxidation (MDA and 4-HNE) levels and protein carbonylation (DNP) levels. Representative image of cytosolic protein carbonylation (DNP) levels. Ponceau was used to normalize the data. (**C**) Representative image of Western blot showing the purity of cytosolic and mitochondrial fractions by using actin and VDAC1 levels. Number of replicates = 3. All data are expressed as the mean ± SEM. (*) vs. SD, (§) vs. 16 w, (&) vs. 20 w, (#) vs. 22 w (*p* < 0.05); (§§) vs. 20 w (*p* < 0.01); *p* values were determined using two-way ANOVA. 4-HNE, 4-hydroxy-2-nonenal; DNP, dinitrophenyl; H_2_O_2_, hydrogen peroxide; MDA, malondialdehyde; SD, standard chow diet.

Considering the link between mitochondria and ROS production, we investigated whether the described mitochondrial alterations are associated with ROS production that may accelerate mitochondrial injury during NAFLD progression. Surprisingly, Amplex Red assay revealed no changes in mitochondrial H_2_O_2_ generation in WD16w in comparison with the respective SD group. Interestingly, a progressive decrease in mitochondrial ROS was observed in WD-fed mice starting from the 20th week, being statistically significant starting from the 22nd week. Although mitochondria isolated from livers of animals fed with WD for 20 weeks exhibited a significantly lower level of carbonylated proteins and lipid peroxides versus the respective control, a clear trend of mitochondrial carbonylated proteins and lipid peroxides levels along disease progression was not found (Figure 4A). In parallel, oxidative stress hallmarks were also evaluated in the liver cytosolic fraction. While no significant differences of DNP-carbonylated proteins were found between WD and SD groups, we only found an upward trend of MDA and 4-HNE levels in the WD22w group when compared with respective SD-fed group (Figure 4B). To exclude cross-contamination of studied intracellular fractions, the purity of isolated mitochondrial and cytosolic fractions was evaluated (Figure 4C). Our data showed no significant alterations in the levels of investigated oxidative damage markers in both cytosolic and mitochondrial fractions along NAFLD progression. Indeed, these findings were not affected by a possible low purity of isolated fractions.

### 2.5. WD Provokes Hepatocyte Alterations in Both Mitochondrial and Non-Mitochondrial Antioxidant Defenses

**Figure 5:**
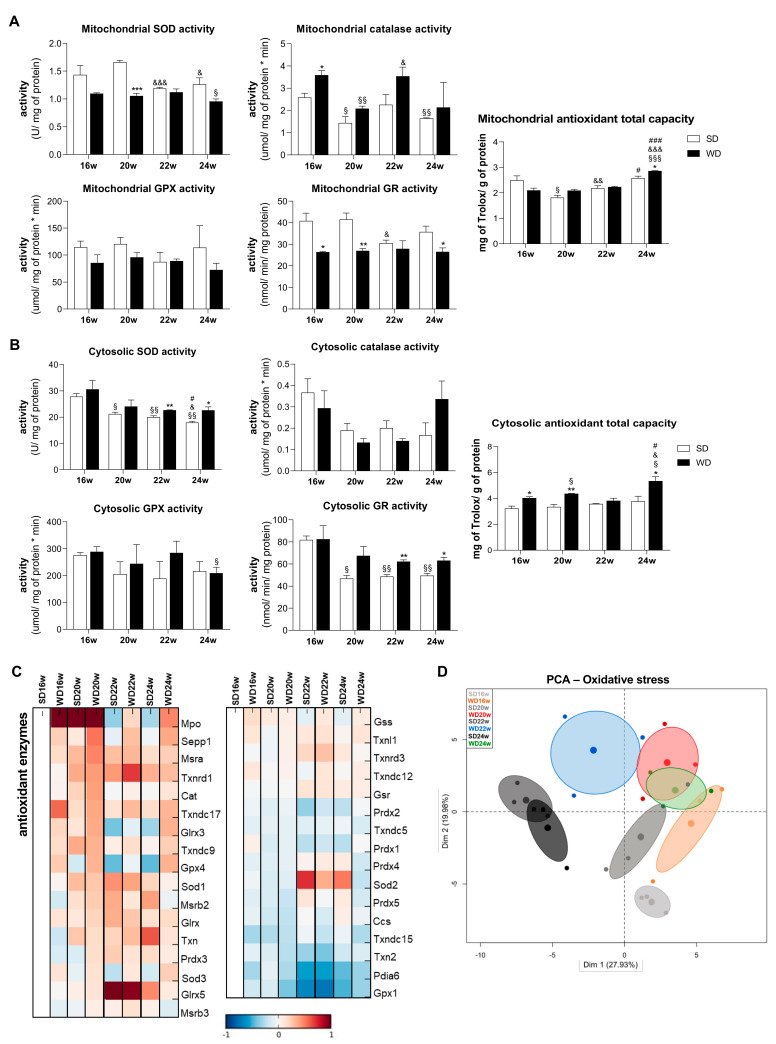
Western diet (WD) causes a hepatic compensatory antioxidant defense response in cytosolic fraction. (**A**) Mitochondrial superoxide dismutase (SOD), catalase, glutathione peroxidase (GPx), glutathione reductase (GR) activities, and mitochondrial antioxidant total capacity. (**B**) Cytosolic SOD, catalase, GPx, GR activities, and cytosolic antioxidant total capacity. (**C**) Mass spectrometry analysis of protein levels involved in the antioxidant defense system. Blue color represents decreased, and red color represents increased levels. (**D**) Principal component analysis (PCA) of oxidative stress-related data. Number of replicates = 3. All data are expressed as the mean ± SEM. (*) vs. SD, (§) vs. 16 w, (&) vs. 20 w, (#) vs. 22 w (*p* < 0.05); (**) vs. SD, (§§) vs. 16 w (*p* < 0.01); (***) vs. SD, (§§§) vs. 16 w, (&&&) vs. 20 w, (###) vs. 22 w (*p* < 0.001). *p* values were determined using two-way ANOVA. SD, standard chow diet.

In order to counteract a negative ROS effect, hepatocytes possess various antioxidant defense mechanisms. In order to investigate them, we analyzed the status of the antioxidant defense system in both mitochondrial and cytosolic fractions. An upward trend of SOD and increased catalase mitochondrial activities were reported in WD16w compared with SD16w. Interestingly, such differences disappear over time. Moreover, we observed a trend for a decrease in GPx activity, while GR activity was significantly decreased in mitochondria for WD16w, WD20w, and WD24w groups. Curiously, mitochondrial TAC was found significantly increased only in the longest time point studied—WD24w group (Figure 5A). In the cytosolic fraction, we found an increase of SOD1 activity, first as a trend for WD16w and WD20w groups, becoming statistically significant from the 22nd week. A similar upward trend was observed for GR activity but in this case from the 20th week. No significant changes were observed in the activities of catalase and GPx, confirming deregulation of the cytosolic antioxidant defense system in WD-fed mice (Figure 5B). Interestingly, the 22nd week of WD feeding coincided with the above-reported cytosolic lipid peroxidation in WD22w group. Additionally, the TAC was significantly increased in livers of WD16w, WD20w, and WD24w groups (Figure 5B). Moreover, MS-based liver proteomic analysis revealed changes of several antioxidant enzymes at the whole tissue level. This analysis revealed higher levels of myeloperoxidase (MPO), selenoprotein P (SEPP1), thioredoxin reductase (TXNRD1), and thioredoxin domain-containing protein (TXNRD17) in all WD-feeding time points tested (Figure 5C). These results are evidence that WD induces oxidative stress mainly in the cytosolic fraction isolated from WD-induced steatotic livers (Figure 5D).

**Figure 6:**
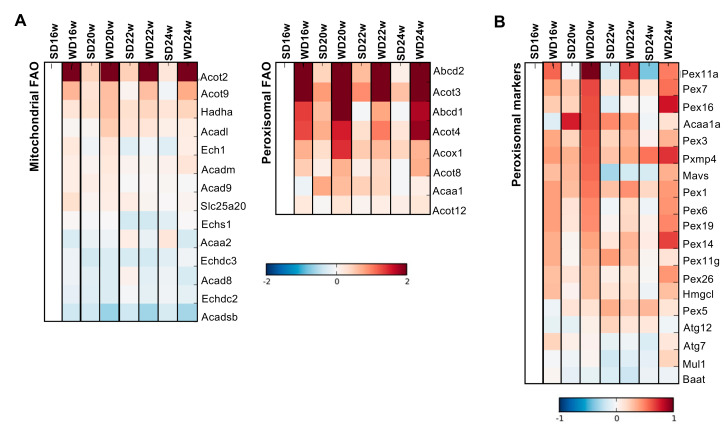
Western diet (WD) feeding is associated with an increment of hepatic oxidative metabolism involving mostly peroxisomes. (**A**) Mass spectrometry quantification of protein levels involved in mitochondrial FAO (left) and peroxisomal FAO (right). (**B**) Mass spectrometry quantification of protein levels of peroxisomal markers. Blue color represents decreased, and red color represents increased levels relative to the SD16w group. Number of replicates = 3. FAO, fatty acid oxidation; SD, standard chow diet; WD, Western diet.

In line with cytosolic lipid peroxidation observations, we investigated the contribution of the peroxisomal-related FAO pathway as a possible ROS source. Even though mitochondrial FAO-related proteins acyl-coenzyme A thiosterase (ACOT)2 and ACOT9 were increased (2-fold and 1-fold, respectively), we found that peroxisomal FAO is the predominant pathway for that event in the livers of WD-fed mice (Figure 6A). Peroxisomal proteins involved in the import of very-long-chain FAs (VLCFAs) (ATP-binding cassette subfamily D member (ABCD)1 and ABCD2), lipid metabolism (ACOT3, ACOT4, ACOT8), and oxidation of fatty acids (acyl-coenzyme A oxidase (ACOX)1) were increased 1- to 2-fold in WD-fed mice from 16th to 24th week compared with SD-fed mice (Figure 6A). Moreover, upregulation of peroxisomal FAO is supported by the increment of peroxisomes number, as demonstrated by augmented levels of peroxisomal proteins involved in biogenesis (PEX1, PEX3), assembly (PEX6, PEX7, PEX26), and membrane import/export (PXMP4, PEX11a, PEX14, PEX16) (Figure 6B). Hence, it seems that FAO contributes to the distinct oxidative metabolism of WD-fed groups compared with SD-fed groups (Supplementary Figure S5D). Altogether, the above-described observations can be the result of an imbalance between peroxisomal-FAO-dependent ROS generation and the cellular antioxidant defense system. Importantly, we demonstrated that WD-associated cellular and mitochondrial alterations have a predominantly non-mitochondrial ROS origin.

### 2.6. WD Increases the Accumulation of Protein-Related Markers of Autophagy: LC3, Sequestosome-1, and Autophagy Cargo-Receptor

**Figure 7:**
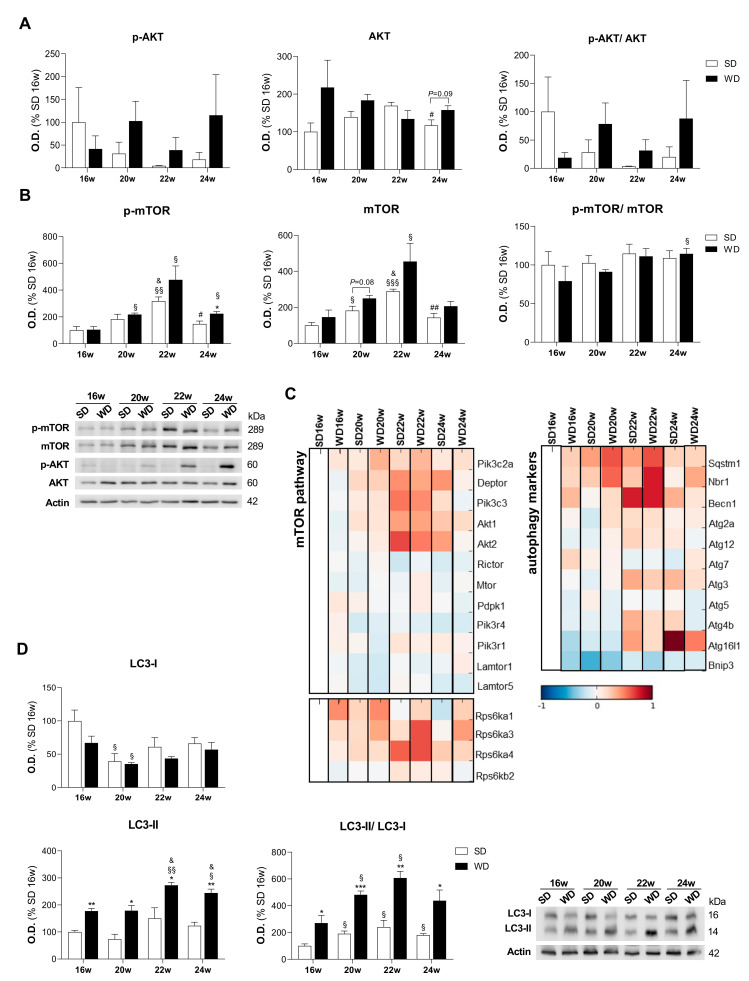
Western diet (WD) is associated with an accumulation of LC3-II and p62 levels in the hepatic tissue. (**A**) Protein level of phosphorylated AKT, total AKT, and p-AKT/AKT ratio. (**B**) Protein level of phosphorylated mTOR, total mTOR, and p-mTOR/mTOR ratio. Representative images of Western blot are shown below. Data are expressed in % of SD16w group. Actin was used as a loading control. (**C**) Mass spectrometry analysis of protein levels involved in mTOR pathway (left side) and autophagy (right side). Blue color represents decreased, and red color represents increased levels relative to the SD16w group. (**D**) Protein level of phosphorylated LC3-I, LC3-II, and LC3-II/LC3-I ratio, with a representative image of Western blot. Actin was used as a loading control. Number of replicates = 3. All data are expressed as the mean ± SEM. (*) vs. SD, (§) vs. 16 w, (&) vs. 20 w, (#) vs. 22 w (*p* < 0.05); (**) vs. SD, (§§) vs. 16 w, (##) vs 22w (*p* < 0.01); (***) vs. SD, (§§§) vs. 16 w (*p* < 0.001). *p* values were determined using two-way ANOVA. SD, standard chow diet.

Intrigued by the alterations in the oxidative stress parameters, we decided to investigate the pathway involved in regulating the cellular adaptative response to the oxidative stress—the PI3K/AKT/mTOR axis. Firstly, we observed no clear trend in the phosphorylation status of AKT at Ser473 and in the p-AKT/AKT ratio in WD20w, WD22w, and WD24w groups compared with the respective controls (Figure 7A). Although significant differences were shown in p-mTOR (Ser2448) in the WD24w group, no clear trend was found in p-mTOR, mTOR, and p-mTOR/mTOR ratio between WD feeding time points and their respective controls over time (Figure 7B). However, MS-based proteomics showed an increased level of proteins involved in mTOR regulation of protein synthesis—RPS6KA1 and RPS6KA3 in all WD-fed groups from the 16th week until the 24th week timepoint (Figure 7C). Moreover, autophagic response, a key cellular quality control mechanism could be influenced by the PI3K/AKT/mTOR axis. While no significant alterations were observed in autophagy-related proteins (ATGs), upregulation of beclin-1 (BECN1) was found in the WD16w and WD20w groups. Then, we also report a progressive increase of 100–150% of LC3-II levels in WD-fed groups over time, together with the consequent increase of 170–360% in LC3-II/LC3-I ratio (Figure 7D). Furthermore, an increased accumulation of 16–18% of sequestosome-1 (SQST1M) and 34–60% of autophagy cargo receptor (NBR1), which recognizes the ubiquitin-modified peroxisomal membrane proteins, was found in all WD-fed groups. Although autophagy was not directly monitored, our results suggest a possible impairment of autophagosomal formation and respective clearance (Figure 7C and Supplementary Figure S5E) in NAFLD progression.

## 3. Discussion

The progression of NAFL into NASH is associated with an adaptative response of hepatocytes and especially mitochondria to the excessive caloric supply. Thus, structural and molecular mitochondrial alterations contribute to a declined mitochondrial function in NASH [6]. In the present work, we revealed the sequence of events that precedes mitochondrial alterations in NAFL development and its progression to NASH. Moreover, we clarified the role of mitochondrial ROS/oxidative stress using a well-characterized mouse model of WD-induced NAFL [16]. Importantly, this study was designed to follow disease progression along time from 16 up to 24 weeks of WD feeding. Here, we demonstrated that WD feeding induced a time-dependent mitochondrial remodeling with augmented respiration, higher protein expression of OXPHOS subunits, and cardiolipin levels at initial NAFL stages, but WD feeding also had a strong impact on decreasing mitochondrial respiration and inducing higher sensitivity to MPTP opening when animals are exposed to WD for longer feeding periods. These events were independent of mitochondrial ROS production, which was found to be decreased in the WD groups. Notably, at the cellular level, impaired levels of autophagic-related markers may contribute to hepatic damage accumulation and NAFLD severity.

High fat and sucrose intake, mimetizing the dietary habits of Western society, is associated with body weight gain and the development of fatty liver in mice with signs of hepatocyte ballooning and early fibrosis along 16, 20, 22, and 24 weeks. Significantly, steatosis is associated with lipid accumulation inside hepatocytes mainly in TGs and cholesteryl esters. By being relatively inert, these lipids provide a protective mechanism of hepatocytes against lipotoxicity-associated damage linked to NASH phenotype [17]. Data show that the staging of NAFLD from the 16th until the 24th week did not significantly vary, either in terms of ballooning or inflammation. Intriguingly, dietary studies that last more than 20 weeks are not often performed. Focusing exclusively on mouse studies, Satapati and colleagues showed that liver steatosis increased continuously along the feeding time, but no liver inflammation was observed until the 32nd week [18]. Therefore, longer WD feeding time points or a second-hit insult would be needed to reach NASH stage.

Growing evidence points to mitochondrial involvement in NAFLD initiation and progression. Mitochondrial deregulation has been described to be critically involved in NAFL/NASH [19], characterized by the loss of mitochondrial ultrastructure, alteration of mitochondrial proteome [7], and the impairment of citrate synthase [12] and OXPHOS activities [20,21]. However, the sequential events of mitochondrial alterations possibly driving NAFLD progression still remained unclear. Importantly, here we showed, for the first time, that NAFLD progression is strongly associated with differential response of mitochondrial respiration. Oxidative phosphorylation was increased at the earlier developmental stage of NAFL (16 and 20 weeks of WD feeding) in comparison with the respective control groups. This finding agrees with augmented levels of OXPHOS subunits belonging to Complex I, II, IV, and ATP synthase at the 16th week of feeding. However, this increased bioenergetic efficiency subsequently showed a progressive decline at the 22nd and 24th weeks (when bioenergetic was supported by Complex II substrate). This initial increase in mitochondrial respiration may be stimulated by nutrient overload. Curiously, in some diet-induced disorders, nutrient overload may cause mitochondrial bioenergetic deregulation, resulting in impaired metabolic flexibility [22]. Indeed, Koliaki et al. showed that obese patients with or without NAFL had higher mitochondrial respiratory rates, while NASH patients, although presenting higher mitochondrial mass, had lower respiratory capacity [6]. Even though it directs comparisons between mice, rat, and humans in terms of the initiation and progression of NAFLD, our results reinforce that there is an early adaptative response of mitochondria to lipids and carbohydrates availability and demonstrate the chronology of mitochondrial events with an increment of mitochondrial capacity preceding a progressive loss of respiratory efficiency along with disease progression. 

Supporting previous research, some authors have reported increased levels of cardiolipin in NAFL and not in NASH [21,23,24]. Indeed, our study also showed increased levels of cardiolipin up to 24 weeks of WD feeding. Cardiolipin is a mitochondrial phospholipid required for OXPHOS complexes assembly and the maintenance of mitochondrial inner membrane fluidity, osmotic stability, and mitochondrial coupling [25,26]. We showed that despite the decline in mitochondrial respiration in NAFL progression, the mitochondria are still functionally coupled, as noted by cardiolipin and RCR values at the 24th week. These data are consistent with Satapati’s study, in which a similar obesogenic diet decreased the RCR in mice after just 32 weeks of feeding [18].

Nevertheless, we demonstrated deleterious effects of fat accumulation at the mitochondrial level over time, as shown by a decreased mitochondrial respiratory efficiency accompanied by a higher susceptibility to calcium-induced mitochondrial permeability. An accumulation of Chol inside hepatocytes seems to have a causal effect on mitochondrial membrane fluidity and dynamics. According to Gan and colleagues, in NASH-affected livers of diabetic mice, mitochondrial Chol deposition activates JNK1-dependent pathway, thereby causing mitochondrial injury with MPTP opening, mitochondrial swelling, and ATP depletion [27]. Moreover, an increase of Chol inside hepatocytes may cause mitochondrial GSH depletion [28,29], associated with a higher hepatocyte susceptibility to inflammatory cytokine TNF-α and Fas sensitization [30]. Thus, both reports showed that Chol accumulation likely contributes to mitochondrial alterations that culminate with hepatocellular apoptosis and the severity of NASH.

Reduced mitochondrial FAO capacity is associated with steatosis and disease progression [7,31]. Interestingly, we did not observe significant alterations in the level of mitochondrial FAO-related proteins. Instead, we found increased peroxisomal markers and peroxisomal FAO-related proteins along WD feeding, suggesting increased peroxisomal mass and peroxisomal FAO to counteract fatty acids overload. These results are consistent with the upregulation of peroxisome proliferator-activated receptor-α (PPAR-α) and ACOX genes, as well as with higher levels of peroxisomal-related proteins in livers of high-fat-fed mice [32,33] and in NAFLD patients [34,35]. In fact, a higher peroxisomal FAO rate was responsible for A/J mouse resistance to diet-induced fatty liver [36]. Accordingly, our data reinforce the emerging role of peroxisomes in hepatic FAO, especially when mitochondrial oxidative capacity is overloaded, as described in high-fat diet-induced steatosis [37]. Although mitochondrial and peroxisomal-oxidative pathways have been associated with an exacerbation of ROS production/oxidative stress [5,38], our previous study, in agreement with Einer et al., did not find increased mitochondrial ROS production or increased mitochondrial-associated oxidative stress in an early NAFL stage [15,16]. This is in sharp contrast with the vast majority of the studies, where it is postulated that mitochondrial ROS generation seems to be involved in the onset of NAFLD. However, in these studies the mitochondrial ROS production and the mitochondrial oxidative damage is not directly monitored [39]. Therefore, in the present study, we demonstrate that mitochondria (a) appear not to participate in ROS production in an early stage of NAFL, and more importantly, (b) the levels of ROS generated by mitochondria exhibit a progressive and significant decrease during NAFLD progression. It has been described that the excessive lipids availability triggers FAO, which in turn, results in increased mitochondrial proton leak and incomplete substrate coupling that may act as a protective strategy against oxidative damage within the mitochondria [40,41]. Interestingly, we found increased OCR values and proton leak-dependent respiration at an early NAFL stage. Therefore, our research shows that mitochondrial ROS production is not implicated in the onset of NAFLD and suggests that mitochondria undergo an adaptive response to avoid inducing hepatic oxidative damage. Additionally, no significant alterations in the activity of mitochondrial antioxidant enzymes was found. These findings are in line with other NAFLD studies lacking evidence to support mitochondria as a primary site for ROS production [12,42,43]. In fact, endoplasmic reticulum and especially peroxisomes were shown to mostly contribute to H_2_O_2_ production in rodent liver [44]. Later on, Elsner and colleagues demonstrated that H_2_O_2_ produced in peroxisomes and not in mitochondria contributes to saturated FAs-induced toxicity in β-pancreatic cells [45]. In line with these data, we recently suggested that peroxisomes are the organelles that contribute to ROS production during the onset of NAFL and disease progression due to their compensatory FAO activity [16]. This is consistent with our observations of increased cytosolic antioxidant enzyme levels and activities (e.g., SOD, GR) and higher cytosolic total antioxidant capacity up to 24 weeks of WD feeding. Although no significant cytosolic oxidative damage was observed, our results suggest that peroxisomal ROS formation constitutes a pro-oxidant stimulus that may contribute to mitochondria sensitization and consequent damage. In fact, in Koliaki’s study, a lower expression of mitochondrial biogenesis regulators (PGC1-α and TFAM) in NAFL and a higher mitochondrial mass in NASH likely indicate an augmented accumulation of damaged and/or dysfunctional mitochondria in severe NASH [6]. This is well correlated with our findings showing an increased accumulation of LC3-II, NBR1, and SQSTM1 along WD feeding up to 24 weeks. Thus, an accumulation of autophagosomes may indicate a time-dependent blockade of the autophagic pathway with consequent accumulation of damaged cellular organelles that might plausibly exacerbate hepatocyte damage and toxicity during NAFLD progression.

In this study, it is of paramount importance to highlight the chronology of the distinct alterations on hepatic and mitochondrial levels that occur along the WD feeding period. Importantly, we have shown the most pronounced effects on liver fat accumulation and hepatocyte damage at the 16th week. From 16th until the 24th week, these hepatic parameters did not significantly change, while a time-dependent remodeling of mitochondrial function was observed. An early augmented mitochondrial respiration was found at the initial NAFL stage, followed by a progressive decreased mitochondrial respiration. Overall, our findings suggest that early cellular alterations that took place until the 16th week might play a critical role in the modulation of NAFLD progression. Then, there are constant accompanying events along with disease progression that may culminate with mitochondrial and hepatocyte collapse. Our work lays the foundations for the mechanisms affected and involved in the early-stage progression of the disease. Notwithstanding, studies with longer periods of WD feeding should be performed in order to reach a more advanced stage of the disease and to uncover whether the alterations described here might have strong impact of NAFLD progression.

In summary, in the present study, we observed that an intake of fat and sucrose-enriched diet for 24 weeks causes the development of NAFL and the initiation of NASH progression in mice hepatic tissue. Our work proves mitochondrial remodeling associated with the progression of the disease, mainly characterized by increased mitochondrial respiration up to the 20th week and a progressive decrease of mitochondrial respiratory efficiency for longer WD feeding timepoints. These observations are accompanied by increased Chol and cardiolipin levels and a higher susceptibility to calcium-induced MPTP opening. Our work reveals that mitochondrial ROS did not trigger such mitochondrial alterations. The most relevant finding is that mitochondrial ROS are not the first “hit” and the first cause of NAFLD progression. Moreover, we have shown that an increased peroxisomal abundance and peroxisomal FAO may counteract lipid accumulation at the expense of cytosolic ROS production and hepatocyte damage. Consequently, accumulation of damaged/malfunctioning organelles, together with a de-regulation of autophagic protein markers, may be determinants to induce the mitochondrial dysfunction and hepatocyte injury that hasten NASH progression.

## 4. Materials and Methods

### 4.1. Materials

All chemicals and reagents used were purchased from Sigma-Aldrich (St. Louis, MO, USA), except those for which another provider is indicated. 

### 4.2. Ethics

The animal studies were approved by the animal welfare regulations of the University of Coimbra and by the Portuguese authority for food and veterinary management (DGAV) (ORBEA_131_2016/24032016 and 0421/000/000/2016, respectively). All the procedures were performed following Directive 2010/63/EU of the European Parliament by certificated operators.

### 4.3. Animals

Four-week-old male C57BL/6J mice purchased from Charles River Laboratories (Charles River, Barcelona, Spain) were housed at the Animal House of the University of Coimbra—UC Biotech with 12 h:12 h light-dark cycles under controlled temperature (22 ± 2 °C) and humidity (55 ± 10%) conditions. After a one-week acclimatization period, mice were divided into eight groups, being four groups fed with standard chow diet (SD) and the other four groups fed with the “Western diet” (WD) in an ad libitum regimen. SD was constituted by 48% carbohydrate, 14% protein, and 4% fat (2014S, ENVIGO Teklad, Madison, MI, USA), and the WD was composed by 35% carbohydrate, 21% protein, and 30% fat (E15126—Ssniff, Soest, Germany), supplemented with 30% (*w/v*) sucrose in the drinking water. The animals were fed with SD or with WD for: 16 weeks (SD16w + WD16w), 20 weeks (SD20w + WD20w), 22 weeks (SD22w + WD22w), and 24 weeks (SD24w + WD24w). The group of mice SD16w was defined as the control group of the study. After the feeding period, animals were fasted and then, anesthetized by isoflurane inhalation, and blood samples were collected by cardiac puncture, followed by animal euthanasia through cervical dislocation. Blood was collected into EDTA KE-coated microtubes (Sarsted, Nümbrecht, Germany), and after being centrifuged, plasma was stored at −80 °C for future analysis. The liver was excised, weighed, and washed with chilled PBS before its division for fixation with paraformaldehyde, for fresh mitochondrial isolation, or storage (−80 °C) until further use. Frozen liver and mitochondrial samples were analyzed at Laboratory of Mitochondrial Biology and Metabolism at Nencki Institute of Experimental Biology (Warsaw, Poland).

### 4.4. Plasma Analysis

For determination of liver damage, alanine aminotransferase (ALT) (ref. A-R0200001001, I.S.E. S.r.l., Guidonia, Italy) and aspartate aminotransferase (AST) (ref. A-R0200001101, I.S.E. S.r.l.) activities were measured according to the manufacturer’s protocol, using a fully-automated analyzer Miura 200 (I.S.E. S.r.l.). 

### 4.5. Histology

For hematoxylin and eosin (H&E), Masson trichrome, and immunohistological stainings, livers were fixed with 10% neutral buffered formalin (HT 50-1-1, Sigma-Aldrich) for 2 days. Next, livers were trimmed and embedded in paraffin wax according to the following protocol: 70% ethanol (two times for 1 h); 80% ethanol (for 1 h); 95% ethanol (for 1 h); 100% ethanol (three times for 1.5 h), xylene (three times for 1.5 h), and finally paraffin wax (two times, at 58–60 °C for 2 h).

(a) For H&E, Masson trichrome and/or immunohistochemistry, paraffin blocks were cut, and liver slices were prepared with 3 µm thickness on SuperFrostPlus microscope slides (Gerhard Menzel GMBH, Braunschweig, Germany). Then, liver slices were deparaffinized and rehydrated before the staining with H&E and Masson Trichrome, which was done according to the following Szymanska-Debinska protocol [46]. In the case of Masson Trichrome, staining included Bouin’s solution (HT10132, Sigma-Aldrich), Weigert’s iron hematoxylin solution (HT1079-1SET, Sigma-Aldrich), and Masson Trichrome Stain kit (HT15-1KT, Sigma-Aldrich).
(b) For immunohistochemistry of CD3, CD45, and CD68, paraffin-embedded liver slices were used for antigen retrieval by applying low pH Target Retrieval Solution (DAKO, Glostrup, Denmark) at 99.5 °C for 30 min. Next, liver slices were incubated with: CD3 (A0452, Agilent, Santa Clara, CA, USA), CD45 (GTX65913 GeneTex, Alton Pkwy Irvine, CA, USA), and CD68 (GTX37743, GeneTex, Alton Pkwy Irvine, CA, USA).
(c) Histological and immunohistochemical stainings were scanned using a Hamamatsu NanoZoomer 2.0 RS scanner (Hamamatsu Photonics, Hamamatsu, Japan) with an original magnification of 40×.
(d) NAFLD activity (NAS) score was determined based on H&E and Masson Trichrome stainings, in which were evaluated four criteria: steatosis (grade 0–3), hepatocellular ballooning (grade 0–2), lobular inflammation (grade 0–3), and fibrosis (S 0–4) [47]. This evaluation was performed in a blind way by the pathologist from The Children’s Memorial Health Institute’s pathologist.

### 4.6. Mitochondria Isolation

Fresh mouse liver (without gallbladder) was washed twice with chilled phosphate buffered saline (PBS) 1× solution and homogenized in an ice-cold buffer I (50 mM Tris-HCl, pH 7.4, 225 mM mannitol, 75 mM sucrose, 0.5 mM EGTA, and 0.5% essentially free bovine serum albumin (BSA)) with a Teflon potter Elvehjem. Then, liver homogenate was centrifuged for 3 min at 740× *g*, 4 °C. The resulting supernatant was again centrifuged for 5 min at 740× *g*, 4 °C. Then, the supernatant was collected and centrifuged for 10 min at 10,000× *g*, 4 °C. Next, the supernatant that contained cytosolic fraction was collected while the mitochondrial pellet was resuspended in buffer II (50 mM Tris-HCl, pH 7.4, 225 mM mannitol, 75 mM sucrose, and 0.5% essentially FAs-free BSA) and centrifuged again for 10 min at 10,000× *g*, 4 °C. The resulting pellet was resuspended with buffer III (50 mM Tris-HCl, pH 7.4, 225 mM mannitol, 75 mM sucrose) and kept on ice up to 3 h. The mitochondrial protein content was determined with the bicinchoninic acid (BCA) assay, using BSA as a standard.

### 4.7. Evaluation of Oxygen Consumption Rates

Oxygen consumption rates (OCR) were measured using a Seahorse XFe96 Extracellular Flux Analyzer (Agilent Scientific Instruments, CA, USA) [48]. On the day before the experiment, a XFe96 sensor cartridge was left to incubate with calibration buffer in a calibration plate at 37 °C. On the day of the experiment, 2.5 µg of freshly isolated liver mitochondria was resuspended in mitochondrial assay buffer (MAS) 1× (2 mM HEPES, pH 7.2, 70 mM sucrose, 220 mM mannitol, 10 mM KH_2_PO_4_, 5 mM MgCl_2_, 1.0 mM EGTA, and 0.2% FAs-free BSA) supplemented with succinate (10 mM final concentration) and rotenone (2 µM final). Mitochondria resuspended in the assay solution was added into a 96-well plate, centrifuged at 2000× *g* for 20 min at 4 °C, and incubated for 20 min at 25 °C. For the evaluation of mitochondrial coupling, the XFe96 sensor cartridge was loaded with ADP (4 mM final) into port A, oligomycin (2 µg/µL final) into port B, carbonyl cyanide-p-trifluoromethoxyphenylhydrazone (FCCP) (4 µM final) into port C, and antimycin A (2 µM final) into port D.

For the electron flow assay, 2.5 µg of freshly isolated liver mitochondria was resuspended in mitochondrial assay buffer (MAS) 1× supplemented with pyruvate (10 mM pyruvate final), malate (10 mM final), and FCCP (4 µM final). XFe96 sensor cartridge was loaded with rotenone (2 µM final) into port A, succinate (10 mM final) into port B, antimycin A (2 µM final) into port C, and ascorbate (10 mM final)/N,N,N′,N′-tetramethyl-p-phenylenediamine (TMPD) (100 µM final) into port D. 

Substrates/inhibitors were injected pneumatically when indicated. Data were analyzed using Software Version Wave Desktop 2.6.

### 4.8. Assessment of MPTP Opening

Opening of the mitochondrial permeability transition pore (MPTP) was determined at 540 nm in a Biotek Cytation 3 reader (Biotek Instruments, Winooski, VT, USA). The experiment was performed with the use of 150 µg of freshly isolated hepatic mitochondria per well’s plate in a reaction buffer composed by 225 mM mannitol, 75 mM sucrose, 1 mM KH_2_PO_4_, 3 mM HEPES, 10 µM EGTA, 10 mM succinate, and 2 µM rotenone. The measurement started with the addition of 200 µM tert-butyl hydroperoxide and 150 µm CaCl_2_ (as MPTP inducers). The negative control included the addition of 1 µM cyclosporin-A before MPTP inducers.

### 4.9. Measurement of Mitochondrial ROS Production

ROS production by mitochondria was measured using Amplex red in a Biotek Cytation 3 reader at 560/590 nm excitation and an emission wavelength, respectively. The assay was performed in the presence of 100 µg of freshly isolated mitochondria in a reaction buffer containing 50 mM Tris-HCl, pH 7.4, 75 mM sucrose, 225 mM mannitol, 5 µM Amplex Red, 40 U/mL superoxide dismutase (SOD), and 20 U/mL horseradish peroxidase, supplemented with 10 mM succinate and 2 µM rotenone. 

### 4.10. Lipid Analysis

(a) Extraction of lipids: Lipids were extracted from frozen liver (1.8 mg) and frozen liver isolated mitochondria (500 µg) according to the method described by Bligh and Dyer [49]. Samples were homogenized in 2 chloroform: 1 methanol mixture containing 0.01% butylated hydroxytoluene. Then, homogenized samples were mixed with distilled water and vortexed before being centrifuged at 3000 rpm for 10 min at 4 °C. The resulting two-phase system was separated, and the bottom layer constituting the organic lipidic phase was collected and stored at −20 °C.
(b) Separation of mitochondrial phospholipids: Stored liver mitochondrial organic samples were evaporated under a N_2_ flow at 37 °C. Next, evaporated samples were resuspended in 2 chloroform: 1 methanol mixture and loaded into a thin-layer chromatography (TLC) silica gel 60 plate (Merck, Darmstadt, Germany) that was previously pre-activated in the oven at 110 °C for 90 min. The different phospholipids species were separated in a TLC tank containing the mobile solvent composed by chloroform/methanol/acetic acid/water (50/37.5/3.5/2 (v/v/v/v)) for 2 h. After TLC, the silica plate was soaked in 10% cupric sulfate and 8% phosphoric acid and put again in the oven at 140 °C for 20 min to visualize phospholipids bands. Bands were quantified using Image Studio Lite (version 5.2) (LI-COR Biosciences, Lincoln, NE, USA).
(c) Separation of neutral lipids: Stored liver organic samples were evaporated under a N_2_ flow at 37 °C. Then, evaporated samples were resuspended in 2 chloroform: 1 methanol mixture and loaded into a TLC silica gel 60 plate. Neutral lipids species were separated in a TLC tank containing the mixture heptane/isopropyl ether/glacial acetic acid (60/40/3 (v/v/v)) for 50 min. After TLC running, the silica plate was soaked in a 10% cupric sulfate and 8% phosphoric acid solution, and neutral lipids bands were visualized after 20 min at 140 °C. Bands were quantified using Image Studio Lite (version 5.2).

### 4.11. Assessment of Lipid Peroxidation

The final products of lipids oxidation (aldehyde malondialdehyde (MDA) and 4-hydroxy-2-nonenal (4-HNE)) were determined following the method described by Gérard-Monnier and colleagues [50]. This assay was performed by adding 230 µg of frozen liver cytosol or 460 µg of frozen liver isolated mitochondria to the reaction buffer composed by N-methyl-2-phenyllindole and acid methanesulfonic, which were further incubated at 45 °C for 40 min. Next, samples were cooled down, centrifuged at 10,000× *g* at 4 °C for 5 min, and the supernatant collected. The supernatant absorbance was measured at 586 nm in a plate reader (Infinite M200, Tecan, Männedorf, Switzerland), and a standard curve with known concentrations of MDA and 4-HNE was used to determine nmoles MDA + 4-HNE per gram of protein in the samples.

### 4.12. Assessment of Protein Carbonylation

Protein carbonylation levels in cytosolic and mitochondrial samples were determined according to the protein carbonyl assay kit (ab178020, Abcam, Cambridge, UK). Samples (20 µg) were prepared following the manufacturer’s protocol. Next, proteins were separated in a 10% sodium dodecyl sulfate (SDS)-polyacrylamide gel and transferred to a polyvinylidene difluoride (PVDF) membrane. Membranes were blocked in 5% non-fat dry milk in TBS (50 mM Tris–HCl, pH 8.0, 154 mM NaCl) for 1 h at room temperature (RT) and incubated with anti-dinitrophenyl (DNP) primary antibody overnight (o.n.). After three 5 min washes in TBS-T (TBS containing 0.1% Tween-20), the membranes were incubated with IRDye^®^800CW secondary antibody (LI-COR Biosciences). After three 5 min washes in TBS-T, DNP-derivatized proteins were visualized in an Odyssey infrared imaging system (LI-COR Biosciences). Levels of DNP were normalized using 0.01% Ponceau staining. The quantification of carbonylation protein levels was performed using Image Studio Lite (version 5.2).

### 4.13. Total Antioxidant Capacity

The assessment of total antioxidant capacity (TAC) in cytosolic and mitochondrial samples was performed following an adaptation [51] of the Arnao et al. method [52]. Accordingly, TAC is determined in function of each sample capacity to reduce the availability of the stabilized form of 2,2′-azino-bis (3-ethylbenzothiazoline-6-sulfonic acid) (ABTS) radical. The ABTS radical was formed by combining 50 mM ABTS with the reaction mixture (50 mM phosphate, pH 7.5, 10 mM H_2_O_2_, and 1 mM horseradish peroxidase) in darkness at 4 °C for 4 h. Next, 90–150 µg of cytosol or 150–300 µg of mitochondria fraction was incubated with the stabilized ABTS radical for 15 min. Absorbance was measured from 0 min timepoint (T0) until 15 min timepoint (T15) at 730 nm in a microplate reader (Infinite 200Pro, Tecan). Data are expressed in terms of mg of Trolox per mg of protein. 

### 4.14. Assessment of Antioxidant Enzymes Activities

Cytosol and isolated mitochondria fractions were prepared in ice-cold lysis buffer (50 mM Tris, pH 8.0, 150 mM NaCl, 1% IGEPAL, and 0.5% sodium deoxycholate) containing protease and phosphatases inhibitors cocktail (1861281, Thermo Fisher Scientific, Waltham, MA, USA). After incubation of 30 min on ice, samples were centrifuged at 15,000× *g* for 15 min at 4 °C. SOD activity was measured using a commercial SOD assay kit (ab65354, Abcam) and reading absorbance at 450 nm. Catalase activity was assessed by following the manufacturer’s protocol of catalase assay kit (707002, Cayman Chemical, Ann Arbor, MI, USA) and measuring absorbance at 540 nm. Then, glutathione reductase (GR) and glutathione peroxidase (GPx) activities were analyzed using GR assay kit (703202, Cayman Chemical) and GPx assay kit (ab102530, Abcam), respectively. Both enzyme activities were assessed at an optical density of 340 nm. SOD activity is expressed in U/mg of protein; catalase and GPx activities in µmol/min/mg of protein; and GR activity in µmol/min/mg of protein.

### 4.15. Western Blotting

For livers, approximately 50 mg of frozen tissue was homogenized in phosphate buffer (50 mM NaH_2_PO_4_/Na_2_HPO_4_, pH 7.5, 1 mM NaF, 1 mM Na_3_VO_4_, 100mM NaCl, 0.1% Triton X-100, 1 mM phenylmethylsulfonyl fluoride (PMSF)) containing a cocktail of inhibitors of proteases and phosphatases (1861281, Thermo Fisher Scientific). Then, liver homogenates were centrifuged at 1500× *g* for 6 min at 4 °C. 

Cytosolic and mitochondrial fractions were lysed in ice-cold RIPA buffer (50 mM Tris, pH 8.0, 150 mM NaCl, 1% IGEPAL, 0.5% sodium deoxycholate, 0.1% SDS) also supplemented with inhibitors cocktail (1861281, Thermo Fisher Scientific) for 30 min. Then, cytosolic and mitochondrial fractions were centrifuged at 15,000× *g* for 15 min at 4 °C, and the supernatants were collected.

Protein concentration of all samples was assessed by Bradford method [53], using BSA as a standard. An equivalent amount to 30–75 µg of protein lysates was prepared with Laemmli buffer, denatured in a dry bath at 95 °C for 5 min (exception for anti-OXPHOS primary antibody), and loaded into 7–14% SDS-polyacrylamide gels. Precision Plus Protein Dual Color Standards (#1610374, Bio-Rad) was used as protein ladder. After protein separation on SDS gels, proteins were transferred to PVDF membranes. PVDF membranes were blocked with 5% BSA or 5% non-fat dry milk in TBS for 1 h at RT and incubated with primary antibodies overnight at 4 °C. After three 5 min washes in TBS-T, the membranes were incubated with the respective horseradish peroxidase-secondary antibodies (goat anti-rabbit (1:10,000, ab6721, Abcam) or goat anti-mouse (1:10,000, ab97023, Abcam)) for 1 h at RT. After three 10 min washes in TBS-T, membranes were incubated with ECL Prime Western Blotting System (RPN2232, Sigma-Aldrich), and the proteins of interest were visualized in a GBOX Chemi XT4 system (Frederik, MD, USA) with GeneSys software (version 1.2.5.0). Protein levels quantification was performed using Image Studio Lite (version 5.2), protein levels being expressed in optical density (O.D.) in arbitrary units, being % calculated relative to SD16w group. The protein amount was normalized to β-actin or Ponceau. 

List of primary antibodies used: AKT (#4691, Cell Signaling, Danvers, MA, USA), LC3 (#12741, Cell Signaling), mTOR (#2972, Cell Signaling), p-AKT (Ser473) (#4060, Cell Signaling), p-mTOR (#2971, Cell Signaling), OXPHOS cocktail (ab110413, Abcam), β-actin (A5441, Sigma-Aldrich), GAPDH (#365062, Cell Signaling), and VDAC1 (ab34726, Abcam).

### 4.16. Proteomic Analysis

Liquid chromatography-MS3 spectrometry (LC-MS/MS) was done at the Thermo Fisher Center for Multiplexed Proteomics (Department of Cell Biology, Harvard Medical School, Cambridge, MA, USA). Peptide samples were studied by using a LC-MS3 data collection strategy in an Orbitrap Fusion mass spectrometer (Thermo Fisher Scientific) by an experienced operator.

(a) Quantitative MS analysis. An ice-cold lysis buffer composed by 50 mM Tris, pH 8.5, 8 M Urea, and 1% SDS supplemented with protease and phosphatase inhibitors (Roche, Basel, Switzerland) was mixed with the liver frozen samples. Protein quantification was assessed using a micro-BCA assay (Pierce Biotechnology Inc., Rockford, IL, USA), and a final concentration of 2–8 mg/mL of liver lysates was used for further analyses. After reduction and alkylation of proteins, proteins were precipitated with methanol (4:1; solvent:protein), chloroform (1:1), and distilled water (3:1). After vortex, the mixture was centrifuged for phase separation, and the aqueous phase containing proteins was collected. Protein extract was washed with ice-cold methanol and dried out. Then, protein extracts were resuspended in 50 mM Tris, pH 8.5, and 4 M Urea solution following its first digestion with LysC (1:50; enzyme:protein) for 12 h. Digested proteins were mixed with 50 mM Tris, pH 8.5, 1 M Urea before their second digestion with trypsin (1:100) for 8 h. The resulting peptides were desalted in a C18 solid-phase extraction cartridge and dissolved in 200 mM EPPS, pH 8.0. At this step, peptides were quantified using the micro-BCA assay (Pierce Biotechnology Inc.). Peptides from each condition were organized in 10-plex samples and labelled with tandem mass tag (TMT) reagent (Pierce Biotechnology Inc.) (1:4; peptide:reagent) for 2 h. The reaction of TMT with tyrosine residues was reconstituted by adding 5% hydroxyl amine for 15 min. Reactions were quenched with 0.5% TFA and samples prepared in 1:1:1:1:1:1:1:1:1:1 ratio for 10-plex samples experiments. Then, samples were desalted and fractionated offline into 24 fractions [54].
(b) LC-MS/MS. Twelve out of twenty-four peptide samples were studied by LC-MS3 data collection strategy [55] on an Orbitrap Fusion mass spectrometer (Thermo Fisher Scientific Inc.) with a Proxeon Easy nLC 1000 dedicated to peptide fractions handling and separation. Peptides (equivalent to 5 µg) were mixed with 5% formic acid and 5% acetonitrile (ACN) solution and loaded into a 100 µm diameter of silica microcapillary that was inserted in a C18 reversed-phase resin (GP118 resin, 1.8 μm, 120 Å, Sepax Technologies Inc., Newark, DE, USA). Peptides factions were analyzed for 2 h in a linear gradient of 3–25% of solvent mixture B (100% ACN and 0.125% formic acid) with solvent mixture A (3% ACN and 0.125% formic acid) at a flow rate of 600 nL/min. Orbitrap Fusion protocol included: MS1 spectrum (resolution of 120,000; 400–1400 m/z scan range; AGC target of 2 × 105; maximum injection time of 100 ms; dynamic exclusion of 75 sec); MS2 (quadrupole isolation set at 0.5 Da and ion trap analysis; AGC, 4 × 103; NCE, 35; maximum injection time, 150 ms) for 2 sec; and MS3 consisting of the top ten precursors of each MS2 scan, which were fragmented by HCD prior to analysis (NCE, 55; AGC, 5 × 104; maximum injection time, 150 ms; isolation window, 2.5 Da; resolution, 60,000).
(c) LC-MS3 analysis. Distinct software was used to evaluate the raw data and control protein false discovery rates and to proceed to the protein quantification of assembled proteins and peptides. Raw data were compared with an UniProt mouse database (2014) (including forward and reverse sequences), and distinct criteria were applied for this analysis: tryptic with two missed cleavages, a precursor mass tolerance of 50 ppm, fragment ion mass tolerance of 1.0 Da, static alkylation of cysteine (57.02146 Da), static TMT labelling of lysine residues and N-terminal of peptides (229.162932 Da), and variable oxidation of methionine (15.99491 Da). TMT tag intensity was quantified using a 0.003 Da range from the theoretical m/z for each reporter tag in the MS3 scan. Poor quality MS3 matches were not considered for the final quantification (<200 summed signal-to-noise across 10 channels, <100 for 6-plex, and <0.5 precursor isolation specificity).
(d) MS data analysis. Protein data (ProteinQuant) for 5930 different protein IDs (SwissProt/UniProt ID) were obtained from the analysis of different plexes (groups of 10 samples in each plex), with the same reference sample per plex. Protein quantification values were normalized by calculating the ratio between the quantification value of the protein in each sample and the quantification value (non-zero) of the protein in the reference sample of the plex. If the quantification value of the protein in the reference sample was zero or null, the final quantification value of the protein in every sample of the plex turned to be null. After excluding zero and null values, a total of 4573 rows corresponding to distinct protein IDs were considered and clustered with the number of significant clusters established by gap statistics. Data (mean between each group and the control group) were integrated in KeggAnim program for visualization of cellular pathways [56], and protein levels were represented as heatmaps by Taverna Workbench Bioinformatics (version 2.5.0).

### 4.17. Statistical Analysis

Data were expressed as the mean value ± standard error of the mean (SEM). Statistical analysis was performed using GraphPad Prism (version 8.0.2) (GraphPad Software Inc., San Diego, CA, USA). The normality of the data was verified using Shapiro–Wilk test. Accordingly, not normally distributed data were analyzed with the nonparametric Kruskal–Wallis test, followed by Mann–Whitney, and normally distributed data were analyzed with the parametric test 2-way ANOVA. The level of significance was as follows: * *p* < 0.05, ** *p* < 0.01, *** *p* < 0.001, for comparations between SD and WD treatments; § *p* < 0.05, §§ *p* < 0.01, §§§ *p* < 0.001 for comparisons between the time of feeding and 16w timepoint; & *p* < 0.05, && *p* < 0.01, &&& *p* < 0.001 for comparisons between the time of feeding and 20 w timepoint; # *p* < 0.05, ## *p* < 0.01, ### *p* < 0.001 for comparisons between the time of feeding and 22 w timepoint.

## Data Availability

The data that support the findings of this study are available from the corresponding authors, [Y.P. and M.R.W.], upon reasonable request.

## Supplementary Materials

The following are available online at https://www.mdpi.com/article/10.3390/ijms22136848/s1, Figure S1: Western diet (WD) induces an increase of liver size with a more pale color up to 24 weeks of feeding; Figure S2: Western diet (WD) induces steatosis, ballooning and early signs of fibrosis but not lobular inflammation in hepatocytes up to 24 weeks of feeding; Figure S3: Western diet (WD) induces a distinct neutral lipid accumulation profile; Figure S4: Western diet (WD) induces higher levels of phosphatidylinositol (PI) up to 22 weeks, in comparison with the standard chow diet (SD); Figure S5: Hepatic mitochondria isolated from Western diet (WD)-fed mice has higher susceptibility to mitochondrial permeability transition pore (MPTP) opening.
