# Supplementary material for: The Alterations of Mitochondrial Function during NAFLD Progression—An Independent Effect of Mitochondrial ROS Production
Source: Int J Mol Sci. 2021 Jun 25;22(13):6848. doi: 10.3390/ijms22136848 (PMC8268944; doi:10.3390/ijms22136848)
Supplement: Supplementary file 1 [file ijms-22-06848-s001.zip › ijms-1248607-supplementary.pdf]

A

Body weight progression

		Time (weeks)																							
		**1**	**2**	**3**	**4**	**5**	**6**	**7**	**8**	**9**	**10**	**11**	**12**	**13**	**14**	**15**	**16**	**17**	**18**	**19**	**20**	**21**	**22**	**23**	**24**
**16W**	**SD**	106.0 ± 3.2	108.3 ± 2.3	114.8 ± 2.9	120.7 ± 3.8	123.0 ± 2.7	122.4 ± 3.3	128.4 ± 4.1	131.9 ± 4.7	131.9 ± 3.5	137.9 ± 4.5	135.2 ± 2.8	136.0 ± 2.6	139.9 ± 2.2	140.7 ± 2.9	143.5 ± 4.5	142.2 ± 3.3								
	**WD**	105.0 ± 0.1	121.5 ± 4.6	131.7 ± 5.4	135.2 ± 6.3	137.9 ± 6.1	146.8 ± 5.1	158.3 ± 6.8	166.6 ± 6.4	172.9 ± 9.7	184.0 ± 8.7	188.9 ± 10.4	193.0 ± 10.8	200.4 ± 12.3	208.0 ± 11.5	208.3 ± 11.3	212.9 ± 10.2								
**20W**	**SD**	108.0 ± 0.2	115.7 ± 1.5	117.7 ± 2.8	123.3 ± 3.9	128.6 ± 2.9	133.9 ± 3.8	134.0 ± 2.4	140.4 ± 3.2	139.4 ± 3.2	144.3 ± 3.0	143.5 ± 4.8	148.1 ± 3.5	149.0 ± 2.9	151.8 ± 2.7	157.4 ± 2.8	157.7 ± 3.7	155.37 ± 3.4	157.1 ± 3.6	159.6 ± 2.9	153.2 ± 3.3				
	**WD**	117,6 ± 2.0	122.5 ± 2.0	129.7 ± 2.2	133.7 ± 3.8	143.0 ± 3.5	146.4 ± 3.2	144.6 ± 3.1	149.2 ± 9.1	155.3 ± 12.8	163.9 ± 15.3	170.3 ± 15.3	176.9 ± 15.5	184.5 ± 14.4	189.8 ± 15.3	196.0 ± 15.2	203.7 ± 18.1	196.6 ± 16.6	205.8 ± 16.9	210.7 ± 15.2	208.3 ± 17.1				
**22W**	**SD**	110.5 ± 2.9	118.0 ± 4.0	121.2 ± 5.7	125.7 ± 6.3	134.7 ± 8.8	139.4 ± 7.8	139.2 ± 6.9	142.0 ± 7.8	143.4 ± 6.5	149.4 ± 6.9	150.7 ± 6.3	154.5 ± 8.0	154.7 ± 8.5	155.5 ± 6.6	164.8 ± 6.4	164.8 ± 7.0	163.0 ± 6.8	164.9 ± 7.5	165.3 ± 8.6	169.6 ± 7.7	174.5 ± 6.8	169.1 ± 5.3		
	**WD**	116.1 ± 4.5	123.5 ± 5,1	132.2 ± 6.6	135.1 ± 7.2	141.9 ± 8.2	148.6 ± 7.1	149.5 ± 8.3	155.1 ± 12.2	161.7 ± 14.5	169.5 ± 15.4	174.7 ± 16.6	177.9 ± 13.2	183.0 ± 12.9	186.7 ± 12.5	192.0 ± 12.1	197.7 ± 13.5	199.2 ± 12.8	200.8 ± 10.8	204.4 ± 9.3	205.1 ± 9.8	207.1 ± 8.4	195.3 ± 7.8		
**24W**	**SD**	98.6 ± 1.2	110.4 ± 2.3	117.2 ± 2.9	121.3 ± 2.0	128.1 ± 2.3	136.1 ± 2.8	138.5 ± 3.4	138.8 ± 2.1	141.5 ± 2.2	142.7 ± 2.5	150.4 ± 2.8	149.0 ± 2.3	153.3 ± 3.5	153.6 ± 2.2	157.9 ± 3.7	159.9 ± 2.4	163.8 ± 3.1	165.8 ± 2.6	166.6 ± 1.2	167.0 ± 1.2	169.0 ± 1.5	170.2 ± 2.5	173.8 ± 1.6	165.8 ± 3.6
	**WD**	112.1 ± 2.3	123.7 ± 3.3	129.6 ± 2.0	136.6 ± 1.8	143.0 ± 1.0	150.4 ± 2.8	153.0 ± 3.4	153.0 ± 1.7	155.0 ± 2.7	158.7 ± 4.0	170.2 ± 2.0	172.7 ± 4.4	179.1 ± 5.2	184.9 ± 6.0	190.2 ± 6.2	198.5 ± 7.1	204.3 ± 6.9	201.1 ± 4.3	204.0 ± 7.3	206.7 ± 4.3	209.5 ± 1.8	213.7 ± 2.4	216.6 ± 3.2	210.1 ± 1.9

B

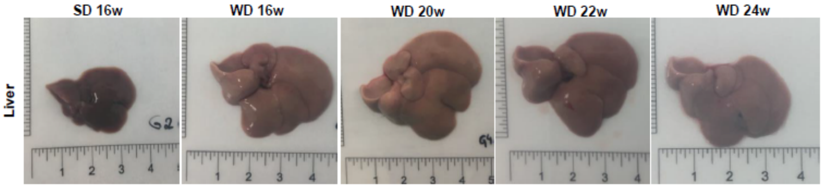

Figure S1. Western diet (WD) induces an increase of liver size with a more pale color up to 24 weeks of feeding. (A) Body weight progression along 16, 20, 22 and 24 weeks of WD feeding. (B) Representative images of liver appearance at the euthanasia time of mice fed a standard chow diet (SD) for 16 weeks and with WD for 16, 20, 22 and 24 weeks. Data are expressed as the mean ± SEM, in grams.

A

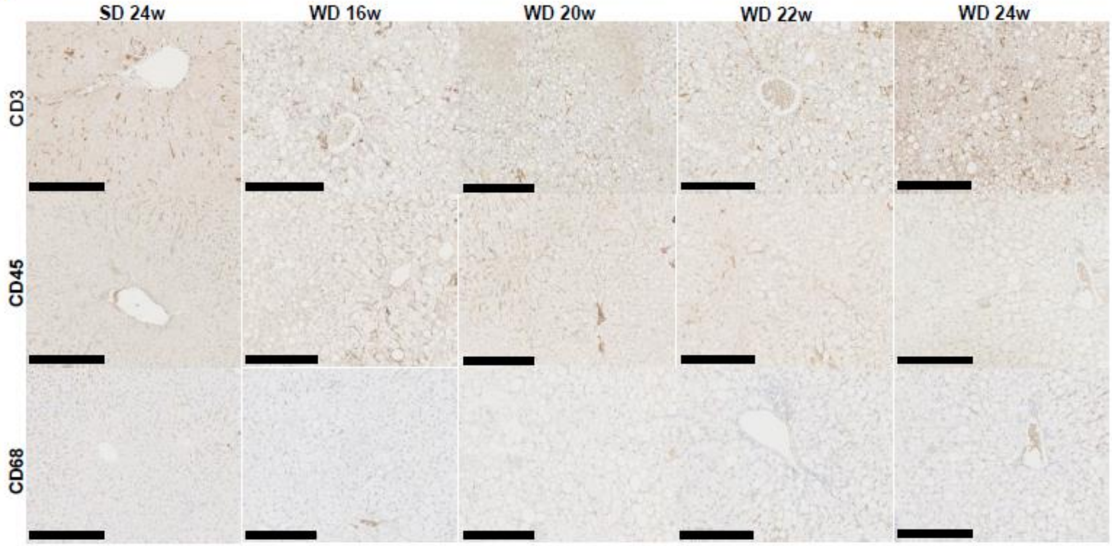

B

NAFLD Activity Score						NAS value[&]
Groups		Steatosis	Balooning	Inflammation	Fibrosis	
**16w**	**SD**	Grade 0	Grade 0	Grade 0	S 0	0
	**WD**	Grade 3 mixed	Grade 2	Grade 0	S 1c	5
**20w**	**SD**	Grade 0	Grade 0	Grade 0	S 1c	1
	**WD**	Grade 2	Grade 2	Grade 0	S 1c	5
**22w**	**SD**	Grade 0	Grade 0	Grade 0	S 1c	1
	**WD**	Grade 3	Grade 1	Grade 0	S 1c	4
**24w**	**SD**	Grade 0	Grade 0	Grade 0	S 0	0
	**WD**	Grade 3	Grade 2	Grade 0	S 1c	5

Figure S2. Western diet (WD) induces steatosis, ballooning and early signs of fibrosis but not lobular inflammation in hepatocytes up to 24 weeks of feeding. (A) Representative images of liver sections with immunohistochemistry of CD3, CD45 and CD68 inflammatory markers. Scale bar: 250 µm. Magnification: 10x. (B) NAFLD activity score (NAS) calculated based of Hematoxylin & Eosin (H&E), Masson Trichrome and immunohistochemistry of CD3, CD45 and CD68 stainings. Steatosis grade: 0 ≤ 5%; grade 1 = 5-33%; grade 2 = 34-66%; grade 3 ≥ 66%. Balooning grade: 0 = absent; 1 = a few ballooned hepatocytes; 2 = many ballooned hepatocytes. Inflammation grade (magnification 200x): 0 = absent; 1 = up to two foci per field of view; 2 = two to four foci per field of view; 3 = more than four foci per field of view (lipogranulomas are counted in this category). Fibrosis grade: S0 = absent; S1a = zone 3, perisinusoidal fibrosis; S1b = zone 3, perisinusoidal fibrosis; S1c = only periportal/ portal fibrosis; S2 = zone 3, plus portal/ periportal fibrosis; S3 = zone 3, plus portal/ periportal fibrosis with bridging fibrosis; S4 = cirrhosis. NAS value& is considered as the sum of the grades obtained for steatosis, ballooning, inflammation and fibrosis parameters. H&E,; SD, standard chow diet.

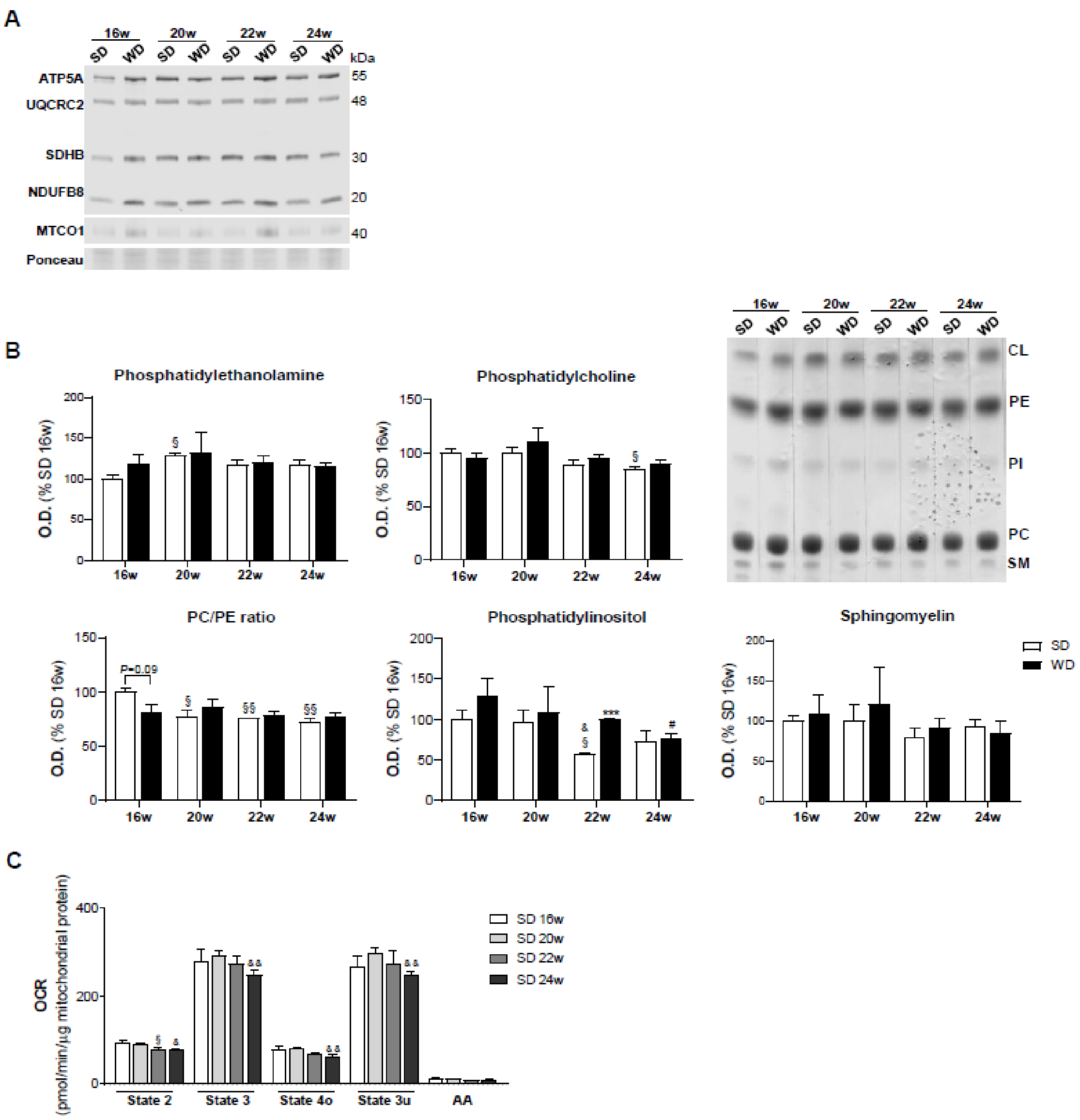

Figure S3. Western diet (WD) induces higher levels of phosphatidylinositol (PI) up to 22 weeks, in comparison with the standard chow diet (SD). (A) Representative image of OXPHOS subunits Western blot. (B) Phosphatidylethanolamine (PE), phosphatidylcholine (PC), PC/ PE ratio, phosphatidylinositol (PI), and sphingomyelin (SM) levels; and representative image of thin layer chromatography. (C) Oxygen consumption rate (OCR) of isolated hepatic mitochondria from SD-fed mice at different time points using succinate as energizing substrate. All data are expressed as the mean ± SEM. (*) vs. SD, (§) vs. 16w, (&) vs. 20w, (#) vs. 22w (P < 0.05); (**) vs. SD, (§§) vs. 16w (P < 0.01); (***) vs. SD, (§§§) vs. 16w (P < 0.001). P values were determined using two-way ANOVA. AA, antimycin A; SD, standard chow diet.

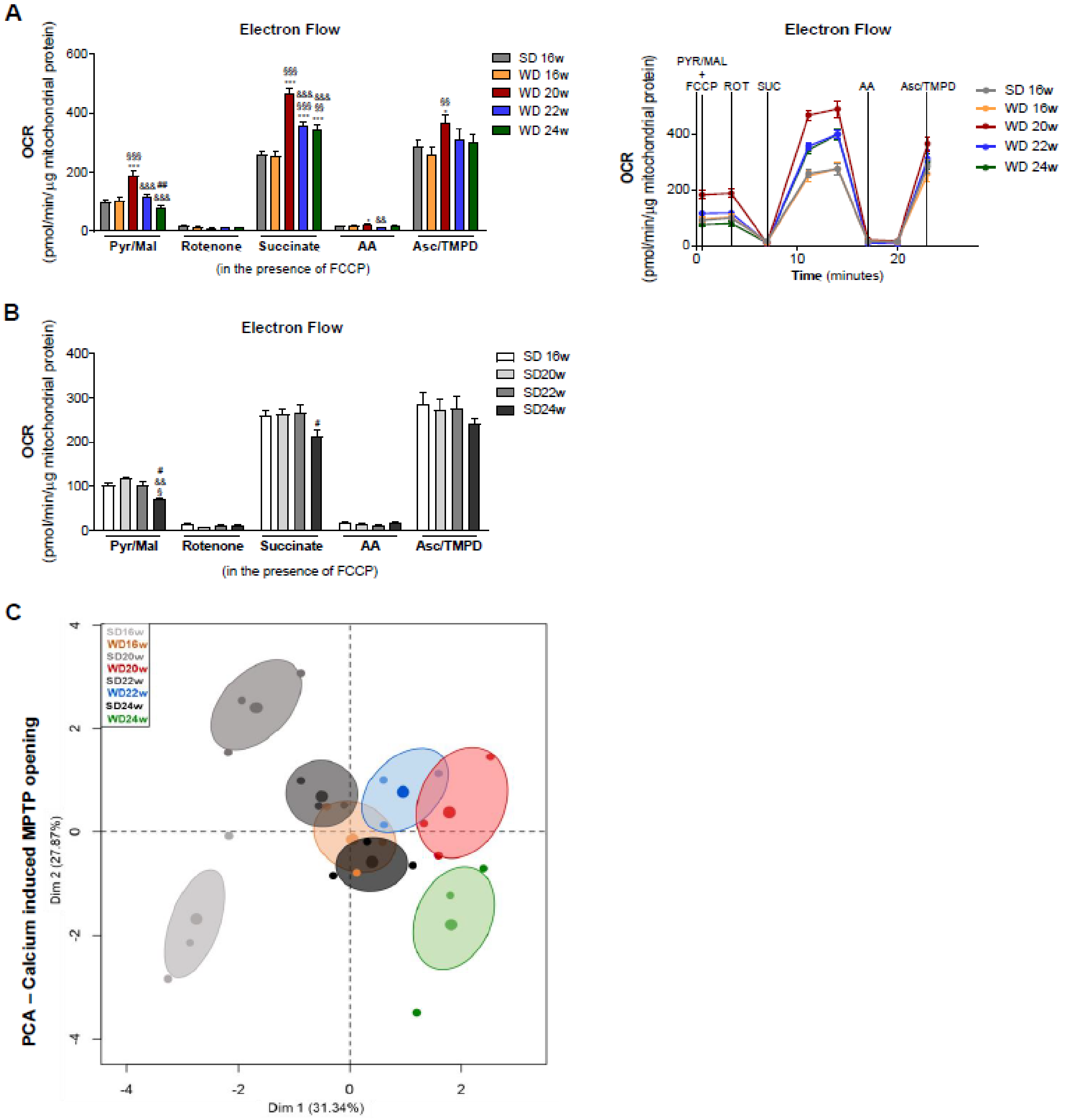

Figure S4. Hepatic mitochondria isolated from Western diet (WD)-fed mice has higher susceptibility to mitochondrial permeability transition pore (MPTP) opening. (A) Electron flow of isolated mitochondria from standard chow diet (SD)-fed mice at 16 weeks and WD-fed mice at different time points (left panel) and the representative profile (right panel). (B) Electron flow of isolated mitochondria from SD-fed mice at different time points. (C) Principal component analysis (PCA) of calcium-induced MPTP opening. All data are expressed as the mean ± SEM. (§§) vs. 16w, (&&) vs. 20w, (##) vs. 22w ($P < 0.01$); (§§§) vs. 16w, (&&&) vs. 20w ($P < 0.001$). P values were determined using two-way ANOVA. AA, antimycin A; Asc, ascorbate; FCCP, carbonyl cyanide-p-trifluoromethoxyphenylhydrazone; Mal, malate; OCR, oxygen consumption rate; Pyr, pyruvate; TMPD, N,N,N',N'-Tetramethyl-p-phenylenediamine dihydrochloride.

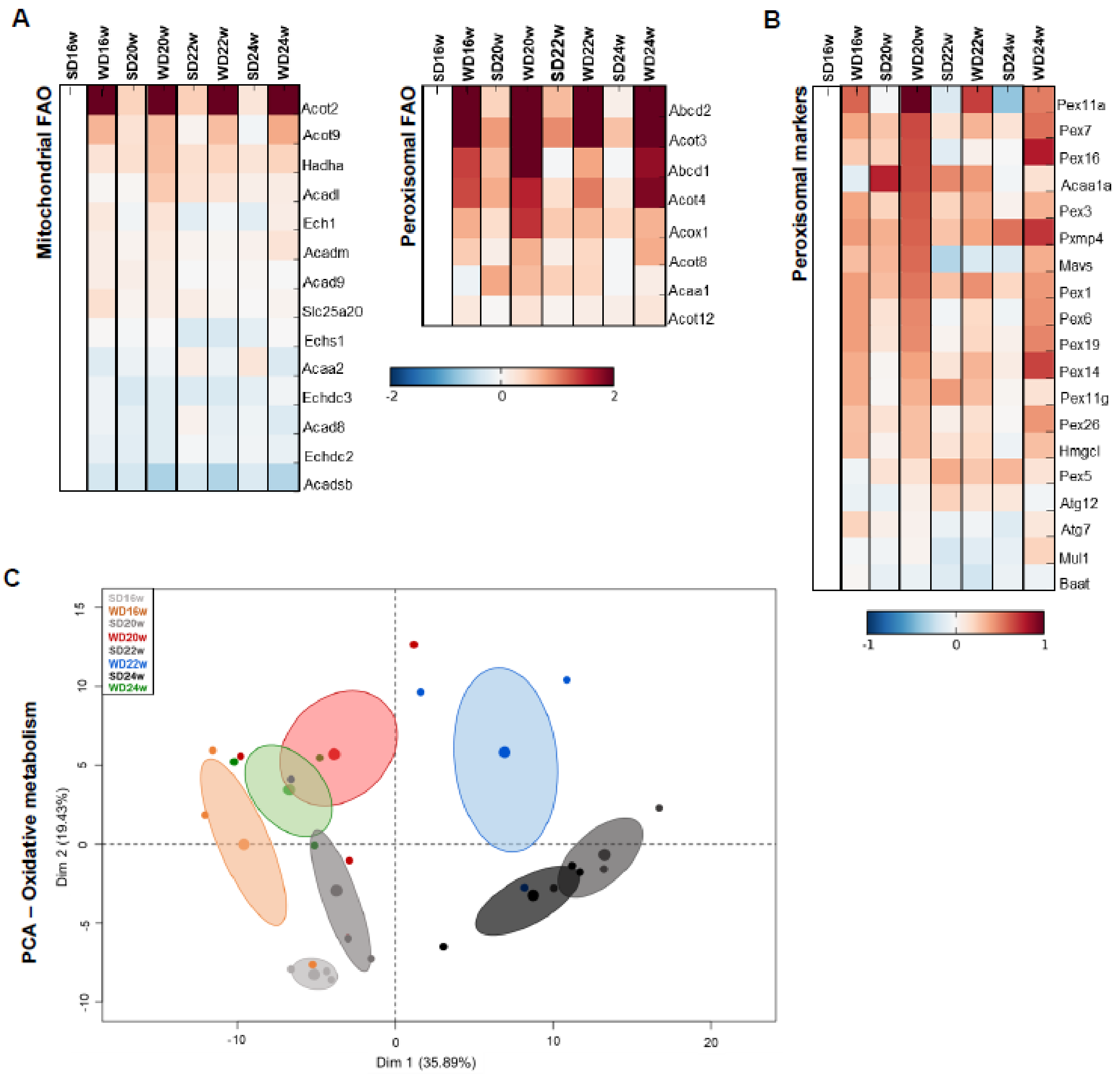

Figure S5. Western diet (WD) feeding is associated with an increment of hepatic oxidative metabolism involving mostly peroxisomes. (A) Mass spectrometry quantification of protein levels involved in mitochondrial FAO (left) and peroxisomal FAO (right). (B) Mass spectrometry quantification of protein levels of peroxisomal markers. Blue colour represents decreased, and red colour represents increased levels relative to the SD16w group. (C) Principal component analysis of oxidative metabolism. FAO, fatty acid oxidation; PCA, principal component analysis; SD, standard chow diet; WD, western diet.

A

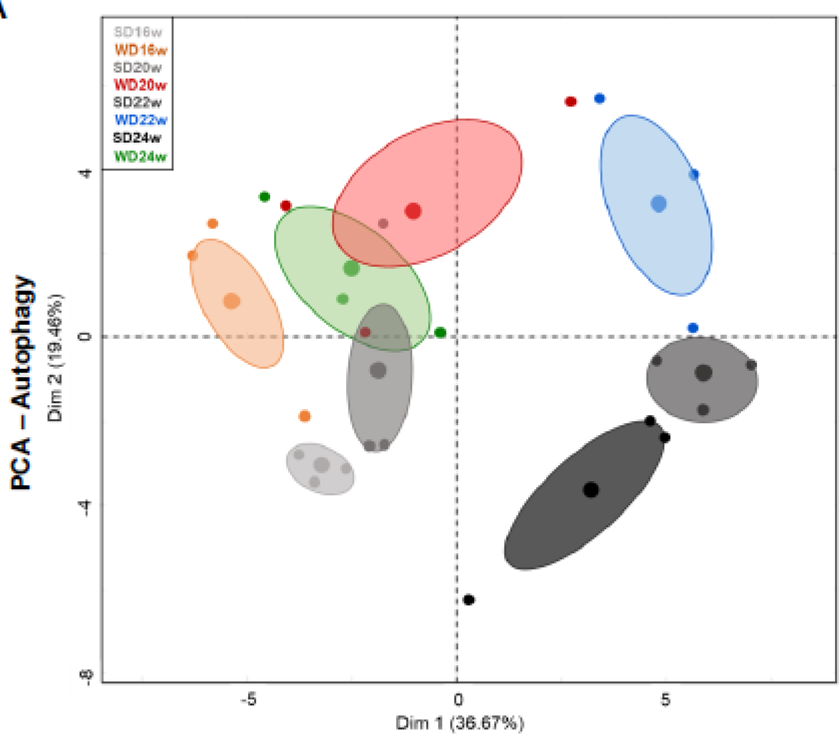

Figure S6. Western diet (WD) feeding induces a distinct hepatic profile of autophagic-related pathways. (A) Principal component analysis (PCA) of autophagy. SD, standard chow diet.
